# Neurobehavioral sex-related differences in *Nf1*^+/−^ mice: female show a “camouflaging”-type behavior

**DOI:** 10.1186/s13293-023-00509-8

**Published:** 2023-04-26

**Authors:** Sofia Santos, Beatriz Martins, José Sereno, João Martins, Miguel Castelo-Branco, Joana Gonçalves

**Affiliations:** 1 Coimbra Institute for Biomedical Imaging and Translational Research (CIBIT), University of Coimbra, Coimbra, Portugal; 2 Institute of Nuclear Sciences Applied to Health (ICNAS), University of Coimbra, Coimbra, Portugal; 3 Faculty of Medicine, University of Coimbra, Coimbra, Portugal

**Keywords:** Autism spectrum disorder, Camouflage behavior, Excitation/inhibition imbalance, Hippocampus, Neurofibromatosis type 1, Sex differences

## Abstract

**Background:**

Neurofibromatosis type 1 (NF1) is an inherited neurocutaneous disorder associated with neurodevelopmental disorders including autism spectrum disorder (ASD). This condition has been associated with an increase of gamma-aminobutyric acid (GABA) neurotransmission and, consequently, an excitation/inhibition imbalance associated with autistic-like behavior in both human and animal models. Here, we explored the influence of biological sex in the GABAergic system and behavioral alterations induced by the *Nf1*^+/−^ mutation in a murine model.

**Methods:**

Juvenile male and female *Nf1*^+/−^ mice and their wild-type (WT) littermates were used. Hippocampus size was assessed by conventional toluidine blue staining and structural magnetic resonance imaging (MRI). Hippocampal GABA and glutamate levels were determined by magnetic resonance spectroscopy (MRS), which was complemented by western blot for the GABA(A) receptor. Behavioral evaluation of on anxiety, memory, social communication, and repetitive behavior was performed.

**Results:**

We found that juvenile female *Nf1*^+/−^ mice exhibited increased hippocampal GABA levels. Moreover, mutant female displays a more prominent anxious-like behavior together with better memory performance and social behavior. On the other hand, juvenile *Nf1*^+/−^ male mice showed increased hippocampal volume and thickness, with a decrease in GABA(A) receptor levels. We observed that mutant males had higher tendency for repetitive behavior.

**Conclusions:**

Our results suggested a sexually dimorphic impact of *Nf1*^+/−^ mutation in hippocampal neurochemistry, and autistic-like behaviors. For the first time, we identified a “camouflaging”-type behavior in females of an animal model of ASD, which masked their autistic traits. Accordingly, like observed in human disorder, in this animal model of ASD, females show larger anxiety levels but better executive functions and production of normative social patterns, together with an imbalance of inhibition/excitation ratio. Contrary, males have more externalizing disorders, such as hyperactivity and repetitive behaviors, with memory deficits. The ability of females to camouflage their autistic traits creates a phenotypic evaluation challenge that mimics the diagnosis difficulty observed in humans. Thus, we propose the study of the *Nf1*^+/−^ mouse model to better understand the sexual dimorphisms of ASD phenotypes and to create better diagnostic tools.

**Supplementary Information:**

The online version contains supplementary material available at 10.1186/s13293-023-00509-8.

## Highlights

- Female *Nf1*^+/−^ exhibit a “camouflaging”-type behavior mimicking autistic girls;
- Female *Nf1*^+/−^ show a more anxious phenotype, but better social and memory performance;
- Female *Nf1*^+/−^ display higher susceptibility to excitation/inhibition imbalance;
- Male *Nf1*^+/−^ display repetitive behavior and develop cognitive deficits;
- *Nf1*^+/−^ mouse model is a good candidate to study female camouflaging effects.

**Supplementary Information:**

The online version contains supplementary material available at 10.1186/s13293-023-00509-8.

## Background

Neurofibromatosis type 1 (NF1) is a common autosomal dominant disorder [1, 2], affecting 1 in 3500 people [3]. About 50% of these patients show a clear inheritance pattern, whereas the rest result from a spontaneous random mutation in the *Nf1* tumor suppressor gene [4, 5]. NF1 patients, among other clinical features [6], shown a high comorbidity with autism spectrum disorders (ASD). The estimated NF1 prevalence among a children population with ASD is approximately 4–5 times higher than NF1 prevalence in general population [7]. Children with NF1/ASD share many similarities to the children with only ASD, such as deficits in communicative skills, difficulty with emotional/social reciprocity and repetitive body movements [7].

Neurofibromin, encoded by the gene *Nf1*, is negative regulator of Ras activity [8, 9]. Previous studies have suggested that hyperactivation of Ras activity leads to long-term potentiation deficits in *Nf1*^+*/−*^ mice causing increased inhibitory neurotransmission [10, 11]. In NF1 patients, we have found that excitation/inhibition (E/I) balance is altered in the visual and medial frontal cortex of patients with NF1 [12, 13]. Moreover, we observed a decreased binding of GABA(A) receptors in patients in the parieto-occipital cortex, midbrain, and thalamus suggesting neurodevelopmental synaptopathy both at the pre- and postsynaptic level [14]. More recently, we found evidence for distinct pre- and postsynaptic phenotypes in the *Nf1*^+*/−*^ mouse model, supporting the E/I imbalance hypothesis but in a region-dependent manner [15].

Similar to other ASDs, NF1 has shown a phenotypical heterogeneity not only due to regional E/I imbalance, but also in relation to biological sex. Although the prevalence of this disorder is similar in men and women, its development and severity vary with sex. Indeed, Diggs-Andrews et al. showed that females tend to develop NF1-associated optic gliomas, while males show more propensity to develop cognitive deficits in both humans and mice [16, 17]. Accordingly, it was demonstrated that there is a significant male bias in the prevalence of ASD in NF1, with greater social communication impairment in males [18]. A recent study has described that at molecular level differences between male and female NF1 also occur [19]. Indeed, microglial cAMP levels were reduced in male *Nf1*^+*/−*^ mice leading to changes in purinergic-mediated microglial phagocytic activity in a sex-dependent manner [19]. Overall, these findings suggested that biological sex is a key factor in the manifestations of *Nf1*^+*/−*^.

Here, we hypothesized that biological sex is crucial to define both NF1-related neurochemical and neurobehavioral profiles. For the first time, we found that female *Nf1*^+*/−*^ mice exhibit a “camouflaging”-type behavior similar to autistic girls. They display a significative E/I imbalance but a better social and memory performance. Mutant juvenile males showed morphological hippocampal changes, impairments in spatial memory and hyperactive-like behaviour.

## Methods

### Animals

**Fig. 1:**
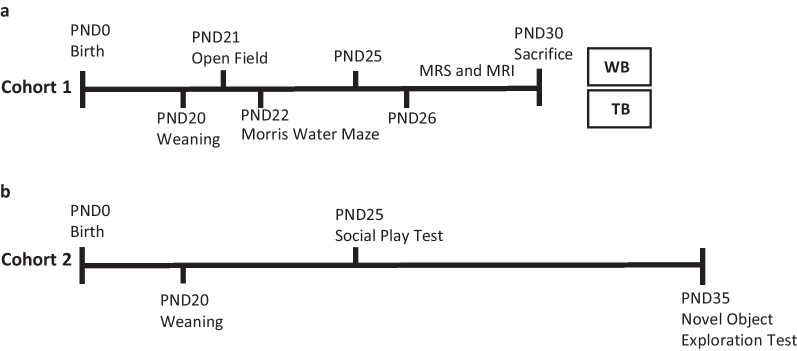
Experimental timeline. **a** Animals in cohort 1 were tested at postnatal day (PND) 21 for open field test and from PND22 to PND25 for the Morris water maze. From PND26 to PND30 animals underwent magnetic resonance imaging and spectroscopy. Further, the hippocampi were used for western blot (WB) and toluidine blue staining (TB) studies. **b** Animals in cohort 2 were tested at PND25 for social play test and PND35 for novel object exploration test

Two cohorts of female and male *Nf1*^+/−^ mice (cohort 1: *n* = 20; cohort 2: *n* = 101; Fig. 1) were obtained from mutant animals C57BL/6N backcrossed only once to 129T2/SvEmsJ in our animal facilities at ICNAS (University of Coimbra) as previously reported [15]. The animals were weaned at postnatal day (PND) 20 and behavioral tests were performed from PND21 to PND25 (cohort 1) and from PND25 to PND35 (cohort 2), to match juvenile age [20]. For cohort 1, animals performed in vivo studies followed by cellular and molecular experiments (Fig. 1a). The brains were extracted and weighted. For cohort 2, the animals were used until PND35 for behavioral tasks (Fig. 1b). Throughout the experiments, WT littermates were used as a control group. Animals were group housed [2-5] on a 12-h light/dark cycle in animal facilities at ICNAS, University of Coimbra. The experiments were carried out in accordance with the European Union Council Directive (2010/63/EU), the National Regulations, and ORBEA board of the ICNAS (1/2017). All experiments were performed and analyzed by blind experimenters. All included animals were healthy (discomfort score 0), and all efforts were made to minimize the number of animals used and their suffering.

### Open field test

The levels of anxiety and exploratory activity of each mouse (PND 21) was measured in white acrylic arena (50 cm *L* × 50 cm *W* × 40 cm *H*), which was equally divided into 16 square areas. The 4 central square areas were considered as the center arena. For the test, each mouse was place in central area and allowed to explore freely for 10 min. The time spent in the central area, total distance, and mean speed was recorded [21].

### Morris water maze test

To evaluate long-term spatial memory, mice performed a Morris water maze (MWM) test at PND22 as previously reported [22]. Mice were trained in a circular pool (120 cm *D* × 60 cm *H*), in a well-lit room with visual cues. The pool water was whitened with non-toxic white dye and the temperature was maintained at 21–23 °C. A clear escape platform (10 cm in diameter) was placed 0.5 cm beneath the water level in the center of a quadrant (north, south, east or west) of the pool in the same location relative to visual cues in the room. Animals were tested for three trials per day over 4 days. Prior to the beginning of testing, mice were allowed to swim freely in the pool for 30 s and then allowed to sit on the escape platform for an additional 30 s. On days 1–4 of testing, the platform was in northwest quadrant for all three trials. Mice were placed in the water from one of the four start positions at the edge of each quadrant and allowed to swim for 60 s. If mice did not find the platform during the allotted time, they were guided toward it, and held for 15 s on the platform. The latency to reach the platform, speed and path length in trials were recorded and measured. Following the third trial, the platform was removed for a probe trail to test spatial memory. During the probe test, mice were allowed to swim for 60 s without the possibility of escape. The time spent in the quadrant, where the platform was previously located was measured.

### Juvenile social-communication play test

Juvenile social behavior was evaluated at PND25 during light period and based on our previous work [23]. Briefly, the day before the test, animals were isolated for 24 h and housed individually into a new cage with fresh bedding. On P25, two animals same-sex, same-genotype animals were placed on a clean, standard cage an anechoic chamber. The vertical walls of the cage were further extended with acrylic sheets to avoid the animal exiting the apparatus. The animals were allowed to interact for 30 min. At the end of the experiment, both animals were returned to their home cage. Video recordings were analyzed by an operator blind to sex and genotype, to identify social behavior. Actions considered as social behaviors included investigative interactions (sniffing, following, mutual circle), affiliative interactions (group sitting, allo-grooming, push under), play solicitation (crawl, push past, approach), and antagonistic interactions (threat, aggression, defense, flight, submission). Total and average social time were manually tracked with a stopwatch, and the number of social interactions was registered. Social preference index, index social preference = (time in social context − time in nonsocial context)/(time in social context + time in nonsocial context) * 100, were compared between groups [24]. At same time with social test, mouse ultrasonic vocalizations (USV) were recorded, to investigate social communication skills. USVs started being recorded as soon as the door of the anechoic chamber was closed and video analysis was performed only from that point on, thus synchronizing acquisition of behavioral data and USV production. USVs recording was performed using a recording system with Avisoft CM16/CMPA condenser microphone placed approximately 10 cm above the top of the test container, UltrasoundGate 416H amplifier and Avisoft Recorder software (Avisoft Bioacoustics, Glienicke/Nordbahn, Germany). Analysis of USVs were done by a blinded operator using the MATLAB toolbox DeepSqueak version 2.6.2, applying the Mouse Call_Network_V2 neural network with a chunk length of 6 s, overlap of 0.1 s, high frequency cut off 125 kHz and no score threshold [25].

### Novel object exploration test

At PND35, during the light period (8:00 AM–11:00 AM), novel object exploration test was performed to explore restricted and repetitive behaviors [26]. Each mouse was placed in a new, filled up to half of its height and empty mouse cage, for a 10 min acclimatization period, and video recorded. Then, four different objects in shape and color, but similar in size—green Lego, red dice, yellow cheese magnet and blue ‘H’ letter magnet—were placed at approximately 3 cm from each corner (Additional file 1: Fig. S1). The behavior of the animal behavior as well as USV production were recorded for 10 min [26]. The position of the objects changed, and all objects were cleaned between trials with an antiseptic. Each position is provided with a number from 1 to 4 and data were analyzed by adapting previous described formulas [27]. A spontaneous alternation (SP) happens when the animal interacts with a different object for every four consecutive interactions and it is calculated by SP = (total sequences of four objects)/(total object interactions-2) * 100. The level of repetitive activity of the animals, using three unrepeated digit sequences, was calculated by sequence repeat index = (most frequent pattern)/(total number of patterns) * 100.

### Video recordings

Videos from the juvenile social play test, empathy test and novel object exploration test, were recorded using a Logitech C170 video camera placed on top of the chamber, while a red light was on. The videos were manually analyzed by a blinded operator. Videos from the open field test and Morris water maze test were recorded using a LifeCam HD-3000 video camera taped to the ceiling of the room recording directly on top of the tests’ site. These videos were analyzed using SMART video-tracking 3.0 software (Panlab/Harvard Apparatus, Spain).

### Magnetic resonance imaging

In vivo image acquisitions were conducted with a 9.4 T magnetic resonance small animal scanner (BioSpec 94/20, Bruker Corporation, Ettlingen, Germany), at the Institute for Nuclear Sciences Applied to Health (ICNAS), University of Coimbra. Animals (between P26 and P30) were anesthetized with isoflurane (delivered through the system E-Z SA800, Euthanex, Palmer, PA, USA), with constant temperature monitoring (Haake SC 100, Thermo Scientific, Waltham, Massachusetts, USA) and assessment of cardiorespiratory function (1030, SA Instruments Inc., Stony Brook, NY, USA).

For volumetric analyses, T2-weighted images were acquired in coronal planes using a RARE sequence: repetition time (TR) = 3600 ms; echo time (TE) = 33 ms; 10 averages; pixel size of 0.078 mm × 0.078 mm and slice thickness of 0.5 mm without spacing between slices (total head 256 pixels × 256 pixels × 32 slices). The analyses of hippocampal volumes were obtained using automatic segmentation feature of ITK-Snap, a software program originally developed at the University of North Carolina, Chapel Hill [28, 29]. Hippocampi were manually traced on the MRI coronal slices by a single expert researcher trained on mouse brain anatomy. Axial, coronal, and sagittal planes were used to ensure anatomical accuracy. Segmentation boundaries were based on existing mouse brain atlas (Allen Reference Atlas—Mouse Brain from atlas.brain-map.org).

### Magnetic resonance spectroscopy

For localized ^1^H-magnetic resonance spectroscopy (^1^H-MRS), data were collected in a volume of interest placed on the hippocampus. B0 maps were acquired before spectroscopy, and shims were optimized through a MAPSHIM voxel. Spectra were acquired using a point-resolved spectroscopy (PRESS) sequence with outer volume suppression (OVS) and VAPOR water suppression [30, 31]. The following parameters were used: TR = 2500 ms, TE = 16.225 ms, number of averages = 720, 3 flip angles = 90°, 142°, 142°, bandwidth = 5000 Hz, number of acquired points = 2048 yielding a spectral resolution of 1.22 Hz/pt. The total acquisition time was 30 min, and voxel size 1.8 * 1.0 * 1.0. Before each spectrum, unsuppressed water spectrum at the same voxel location was acquired (TE = 16.225 ms, TR = 2500 ms, 16 averages, scanning time = 40 s). Data analysis of ^1^H-MRS spectra was performed using linear combination modeling LCModel (Stephen Provencher Inc., Toronto, Canada) [32]. Metabolite quantification was performed applying the internal water reference method. Concentrations in millimole units were calculated for metabolites, and results are presented in arbitrary units (a.u.). Only metabolites with Cramér–Rao bounds < 20% were considered for statistical analysis.

### Brain sectioning

Mice were anaesthetized with intraperitoneal injection (i.p.) of 100 mg/kg ketamine (Nimatek® 100 mg/ml, Dechra) and 10 mg/kg xylazine (Sedaxylan® 20 mg/ml, Dechra) and intracardially perfused with 10 ml of 0.01 M phosphate-buffered saline (PBS) solution (137 mM NaCl; 2.7 mM KCl; 1.8 mM KH_2_PO_4_; 10 mM Na_2_HPO_4_·2H_2_O; pH 7.4) followed by 20 ml of 4% paraformaldehyde (PFA) in 0.01 M PBS, pH 7.4. The brains were removed and post-fixed overnight in 4% PFA solution at 4 °C, followed by immersion in 20% sucrose in 0.01 M PBS over 24 h at 4 °C. Afterwards, the brains were cut into 40-μm coronal sections across the hippocampus on a cryostat (LEICA CM3050 S, Germany) and slices were collected in cryoprotection solution (30% Sucrose; 30% Ethylene glycol; 10 mM phosphate buffer, pH 7.2) until further use.

### Toluidine blue staining

Cryo-sectioned hippocampal slices were mounted onto gelatinized slides and were kept dry at room temperature (RT) overnight. Brain samples were immersed successively in ethanol 100% and 90% for 2 min. Then, slides were stained with toluidine blue solution (77 mM Na_2_HPO_4_·2H_2_O; 67.2 mM citric acid; 3.3 mM toluidine blue) for 10 min. The slices were dipped on a sequential increasing percentage of ethanol, 70%, 90% and 100% for 2 min each, followed by xylene for 5 min, and again 100% and 90% ethanol for 2 min each and toluidine blue solution for 5 min. Finally, they were immersed again in an increasing percentage of ethanol, first 70% for 1 min, followed by 90% for 1.5 min and 100% for 5 min, and xylene 3 × 5 min and left to dry in RT. Brain sections were mounted with DPX mounting medium (Permount™ mounting medium, Electron Microscopy Sciences) and left to dry. Images from hippocampal subregions—CA1, CA3 and dentate gyrus—were acquired on a light microscope (AX10, ZEISS, Germany). For image analysis, 10× magnification images from each hippocampal subregion were selected and layer thickness was measured using FIJI ImageJ 1.8.0 analysis software (NIH, USA).

### Western blot

Mice were sacrificed by decapitation and hippocampi were dissected on ice, to isolate the total, cytosolic and synaptic fraction using Syn-PER Synaptic Protein Extraction Reagent (Thermo Scientific, Pierce Biotechnology, Rockford, USA) according to the manufacturer’s instructions. Protein concentration was determined by the BCA method (Thermo Fischer Scientific; Massachusetts, U.S.A) and stored at − 20 °C until use. Total hippocampal protein (2.5 μg/μl) were separated by electrophoresis on sodium dodecyl sulfate (SDS)–polyacrylamide gel electrophoresis, transferred onto a polyvinylidene difluoride membrane (PVDF; Millipore, Madrid, Spain), and then blocked with 5% non-fat milk in Tris-buffered saline (TBS, in Mm: 200 Tris-Base, 137 NaCl, pH7.6) with 0.5% Tween 20 for 1 h at RT. Afterwards, membranes were probed with the primary antibody rabbit anti-GABA(A) α1 receptor (1:1000; Ref: AGA-001; Alomone Labs, Israel) 1.30 h at RT. Membranes were then washed, incubated for 1 h with alkaline phosphatase-conjugated secondary antibody (anti-rabbit 1:10,000; Ref: 31341; Thermo Fisher Scientific, Massachusetts, U.S.A) and visualized using ECF reagent (RPN5785, GE Healthcare, U.S.A.) on the Typhoon FLA 9000 gel scanner (GE Healthcare, U.S.A.). Immunoblots were reprobed with anti-GAPDH antibody (1:5000; EPR16891, Abcam, Cambridge, UK) for loading normalization, and densitometric analyses were performed using the Image Studio Lite 5.2 analysis software.

### Statistical analysis

Data are expressed as mean values ± SEM. Ordinary two-way ANOVA followed Tukey’s multiple comparisons test was conducted to compare experimental groups using the GraphPad Prism 8.02 (GraphPad Software, Inc., La Jolla California USA). As indicated in the figure legends, data were considered as statistically significant at *p* < 0.05 and, outliers were defined by values within the interval of mean values ± 3 * SD.

## Results

### Male *Nf1*^+/−^ showed increased hippocampal volume

**Fig. 2:**
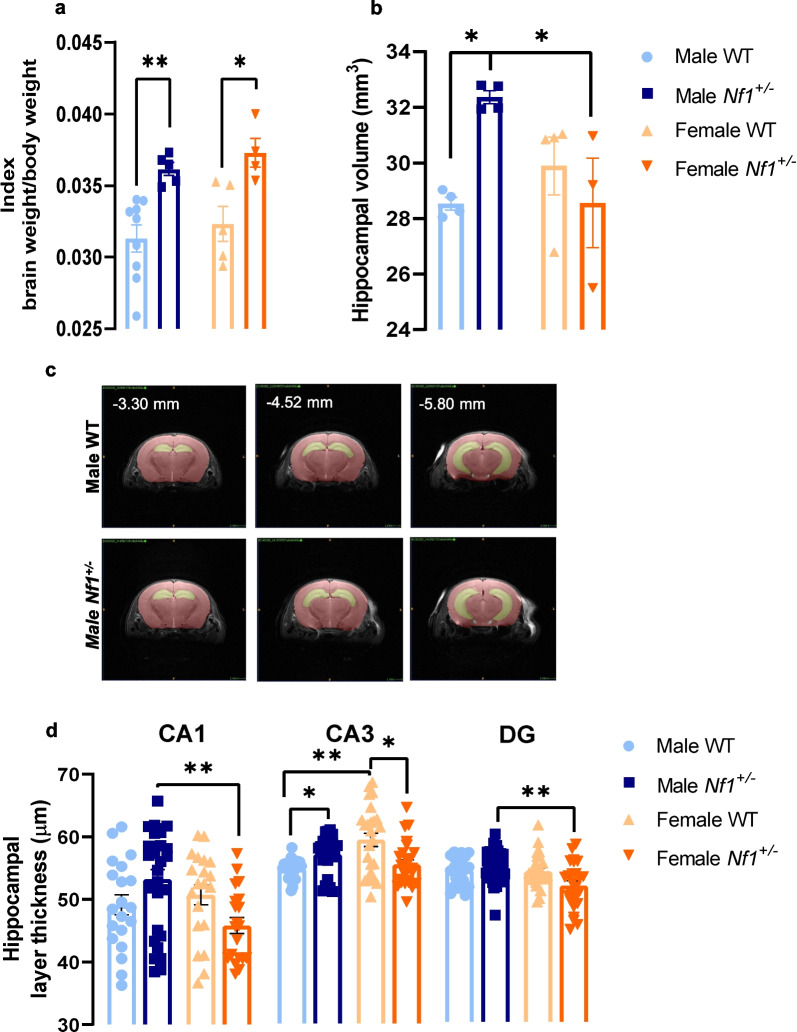
Male *Nf1*^+/−^ mice display hippocampal morphological changes. **a** Both female and male *Nf1*^+/−^ mice showed an increase in index brain weight/body weight (*p < 0.05, **p < 0.01 compared with their littermates WT). **b** Further, transgenic males display higher volume of the hippocampus (*p < 0.05, compared with their littermates WT and with mutant females). Females did not show any differences. **c** Representation of hippocampus mask using in volume measurement by ITK-SNAP software. **d** When we analyzed the thickness of hippocampal subregions, transgenic males exhibited thicker CA1 and DG granule cell layers (**p < 0.01, compared with female *Nf1*^+/−^ mice). Interestingly, CA3 sub-region presented a sexual dimorphism in WT animals (**p < 0.01, compared with female WT mice) that was not observed in *Nf1*^+/−^ mice. Data are expressed as mean ± SEM, *n* = 3–10. Statistical significance was found by two-way ANOVA followed by Tukey’s multiple comparisons test

The WT and transgenic animals were weighted immediately before sacrificed and their brains were freshly extracted and also weighted. We found that there was a significant effect of genotype (*F* (1, 19) = 21.65, *p* = 0.0002). The index brain weight to body weight showed that both male and female *Nf1*^+/−^ mice presented a significant augment in this ratio compared with their WT littermates (male WT: 0.031 ± 0.0010, male *Nf1*^+/−^: 0.036 ± 0.0004, *p* = 0.0095; female WT: 0.032 ± 0.0010, female *Nf1*^+/−^: 0.037 ± 0.0008, *p* = 0.0301; Fig. 2a). Since both animal and human NF1 show hippocampal volume augmentation [33], volumetric analysis of this brain region was performed in both juvenile male and female mice and a clearly interaction sex × genotype was found (*F* (1, 11) = 9.115, *p* = 0.0117). We observed a significantly increase in hippocampus volume only in transgenic male animals compared with their WT littermates (WT: 28.53 ± 0.230 mm^3^, *Nf1*^+/−^: 32.37 ± 0.235 mm^3^, *p* = 0.0177; Fig. 2b). Representative images of hippocampus of neurotypical and transgenic mice are shown in Fig. 2c. We also confirmed the hippocampal morphological changes by performed toluidine blue staining (CA1: *p* = 0.0103; CA3: *p* = 0.0007; DG: *p* = 0.0076). The granule cell layer thickness of sub-regions CA1, CA3 and DG were measured (Additional file 1: Fig. S2) and we found that transgenic male mice showed thicker CA1 and DG than female *Nf1*^+/−^ (CA1—male: 53.17 ± 1.559 µm, female: 45.79 ± 1.297 µm, *p* = 0.0040; DG—male: 62.62 ± 1.055 µm, female: 55.05 ± 1.888 µm, *p* = 0.0028; Fig. 2d). Interestingly, thickness of CA3 displayed sexual dimorphism, whereby female WT showed thicker CA3 than WT male (female: 58.84 ± 2.181 µm, male: 49.53 ± 0.6287 µm, *p* = 0.0016; Fig. 2d). However, this difference was not present in *Nf1*^+/−^ mice suggesting an interaction between sex and the presence of NF1 mutation. In fact, comparing with WT littermates, transgenic male presented an increase in thickness of CA3 (WT: 49.53 ± 0.6287 µm, *Nf1*^+/−^: 54.23 ± 1.096 µm; *p* = 0.0245; Fig. 2d), while the females exhibited the opposite effect (WT: 58.84 ± 2.181 µm, *Nf1*^+/−^: 50.96 ± 1.437 µm, *p* = 0.0125; Fig. 2d).

### Biological sex induced changes in excitation/inhibition balance in NF1 mouse model

**Fig. 3:**
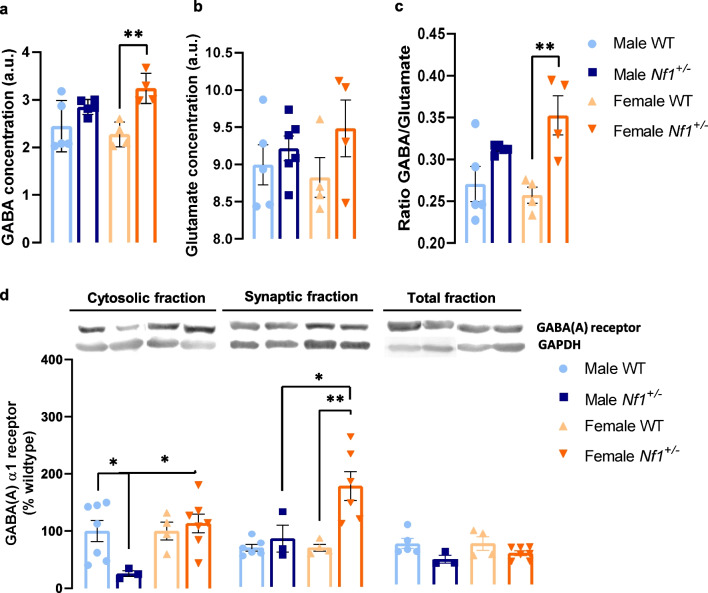
Hippocampal excitation/inhibition imbalance occurs in a sex-dependent manner in *Nf1*^+/−^ animals. **a** Magnetic resonance spectroscopy revealed that mutant females have an increase of GABA concentration (**p < 0.01, comparing with their littermates WT), **b** without changes in glutamate concentration. **c** Accordingly, mutant females display a significant increase in ratio of GABA/glutamate (**p < 0.01, comparing with their littermates WT). **d** Nevertheless, it was found that the *Nf1*^+/−^ mutation leads to a reduction of protein levels of GABA(A) receptor in male cytosolic fraction (*p < 0.05, compared with their littermates WT and with mutant females). On the other hand, transgenic females presented an upregulation of expression of GABA(A) receptor at synaptic fraction (*p < 0.05 and **p < 0.01, compared with mutant males and with WT females, respectively). No changes were detected in total fraction. Data are expressed as mean ± SEM, *n* = 4–7. Statistical significance was found by two-way ANOVA followed by Tukey’s multiple comparisons test

We previously reported that adult *Nf1*^+/−^ mouse exhibited a disproportionate expression of GABA(A) receptors in hippocampal synaptosomes [15]. To understand the impact of biological sex on hippocampal E/I balance in juvenile male and female *Nf1*^+/−^ mice, GABA and glutamate concentration were measured by MRS (Additional file 1: Fig. S3). It was observed a genotype effect on GABA concentration using two-way ANOVA (*F* (1, 14) = 16.45, *p* = 0.0012). An increase of GABA levels was observed in mutant females comparing with their littermate’s WT (female WT: 2.27 ± 0.130 a.u., female *Nf1*^+/−^: 3.24 ± 0.159 a.u.; *p* = 0.0085; Fig. 3a), which leads to an augment of ratio GABA/glutamate (female WT: 0.2573 ± 0.010, female *Nf1*^+/−^: 0.3528 ± 0.023; *p* = 0.0073; Fig. 3c). In this ratio, it was also observed a genotype effect (*F* (1, 14) = 17.75, *p* = 0.0009). However, no changes were found between groups in glutamate concentration (male WT: 8.996 ± 0.2700 a.u., male *Nf1*^+/−^: 9.215 ± 0.1653 a.u. female WT: 8.824 ± 0.2675 a.u., female *Nf1*^+/−^: 9.48 ± 0.3823 a.u.; Fig. 3b). Further, to disclose how hippocampal GABA signaling is influenced by sex under the influence of the *Nf1*^+/−^ mutation, protein levels of GABA(A) receptor were analyzed. We found that expression levels of GABA(A) receptor of male *Nf1*^+/−^ mice were reduced in the cytosolic fraction, compared with their WT littermates (WT: 100.00 ± 18.410%, *Nf1*^+/−^: 25.63 ± 5.091%; *p* = 0.0167; Fig. 3d) and mutant female (113.42 ± 16.098%; *p* = 0.0285; Fig. 3d). On the other hand, and in accordance with GABA levels results, female *Nf1*^+/−^ mice showed an increase in GABA(A) receptor levels in the synaptic fraction when compared with female WT (WT: 100.00 ± 8.611%, *Nf1*^+/−^: 257.70 ± 25.891%; *p* = 0.0446; Fig. 3d) and with mutant male (123.41 ± 34.669%; *p* = 0.0245; Fig. 3d). Interestingly, both data from cytosolic and synaptic fraction showed a sex × genotype interaction (cytosolic fraction: *F* (1, 17) = 5.354, *p* = 0.0335; synaptic fraction: *F* (1, 15) = 5.780,* p* = 0.0296). No changes were detected in the total fraction between groups (Fig. 3d).

### *Nf1*^+/−^ mutation induces sex differences in anxiety manifestations

**Fig. 4:**
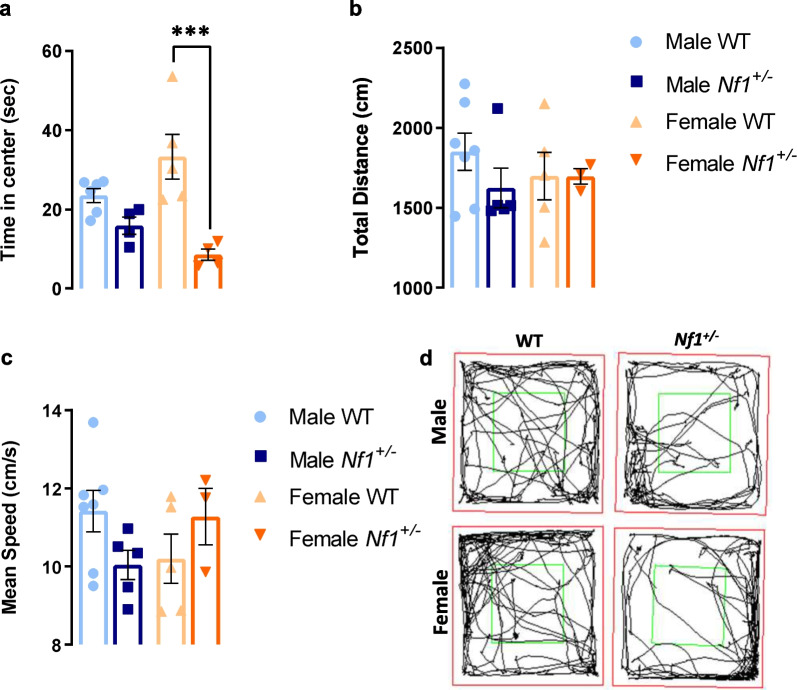
Female *Nf1*^+/−^ mice exhibit more anxious-like behavior. **a** In the open field test, mutant females spent less time in the center of the arena (***p < 0.001, comparing with their littermates WT). **b**, **c** No differences were detected in both total distance travelled and mean speed between groups. **d** Representative images of path length in open field are showed. Data are expressed as mean ± SEM, *n* = 4–7. Statistical significance was found by two-way ANOVA followed by Tukey’s multiple comparisons test

It is known that children with NF1 have anxiety problems [34], but how this characteristic is affected by sex remains unclear. Through the open field test, we observed that female *Nf1*^+/−^ mice display a significantly more anxious-like behavior than WT females by spending less time in the center (WT: 33.320 ± 5.688 s, *Nf1*^+/−^: 8.558 ± 1.464 s, *p* = 0.0008; Fig. 4a). In addition, it was observed an interaction between sex and genotype using two-way ANOVA (*F* (1, 15) = 6.162, *p* = 0.0254). However, no changes were detected in path length (Fig. 4b) neither in mean speed (Fig. 4c). The tracking of male WT and *Nf1*^+/−^ as well as female WT and *Nf1*^+/−^ is represented in Fig. 4d.

### Female *Nf1*^+/−^ exhibit better spatial memory

**Fig. 5:**
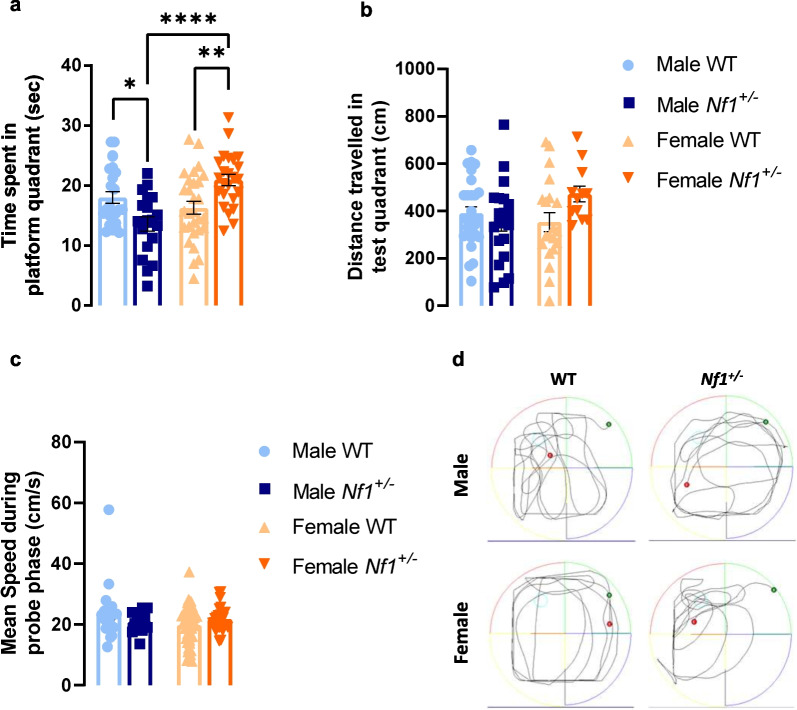
Female *Nf1*^+/−^ mice show surprisingly better spatial memory performance in opposition with the effect found in male *Nf1*^+/−^ mice. **a** Regarding time spent in platform quadrant during probe phase, our results demonstrated that mutant mice showed an impairment in spatial memory (*p < 0.05, comparing with their littermates WT). Interestingly, female *Nf1*^+/−^ mice have better performance by spending more time in this quadrant (**p < 0.01, comparing with their littermates WT; ****p < 0.001, comparing with male *Nf1*^+/−^ mice). **b**, **c** No changes were detected between groups in distance travelled and mean speed during probe phase. **d** Representative image of path length of Morris water maze test. Data are expressed as mean ± SEM, *n* = 3–7. Statistical significance was found by two-way ANOVA followed by Tukey’s multiple comparisons test

Finally, and taking into account prior knowledge that the *Nf1*^+/−^ mouse model presented memory impairment [33], we explored the sex differences in spatial memory of these animals. At the probe phase, we observed a clear sex x genotype interaction according to two-way ANOVA test (*F* (1, 93) = 18.48, *p* < 0.0001). Comparing with their littermates WT, male *Nf1*^+/−^ mice spent less time in platform quadrant (WT: 17.980 ± 0.976 s, *Nf1*^+/−^: 13.590 ± 1.187 s, *p* = 0.0301; Fig. 5a). This impairment becomes even more significant when compared with female *Nf1*^+/−^ (female: 20.940 ± 0.919 s, *p* < 0.0001; Fig. 5a), who showed surprisingly greater memory performance than their littermates WT (WT: 16.270 ± 1.069 s, *p* = 0.0071; Fig. 5a). During probe test, the analyzed groups did not differ in distance travelled and mean speed (Fig. 5b, c). Figure 5d represents the path length of transgenic and WT mice during the probe phase.

### Female *Nf1*^+/−^ animals spent more time in a social context

**Fig. 6:**
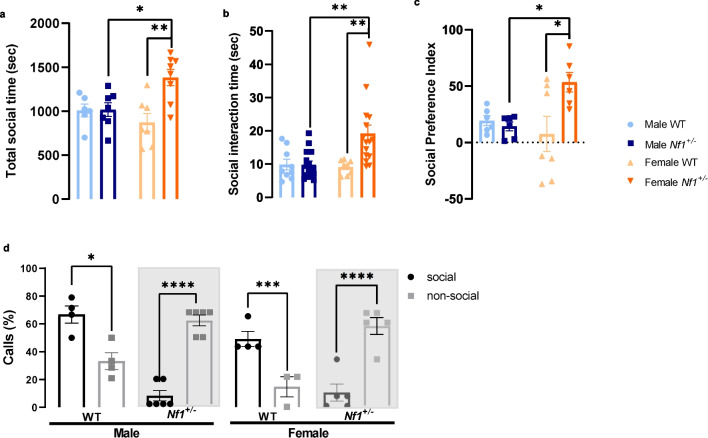
Female *Nf1*^+/−^ display an increase in social behavior. **a** During a juvenile social play test, it was found that female *Nf1*^+/−^ mice spent more time socializing (*p < 0.05 and **p < 0.01, comparing with mutant male and WT female, respectively) and **b** the duration of social interactions was also significantly longer (**p < 0.01, comparing with mutant male and WT female). **c** Accordingly, mutant female showed an increase of social preference index (*p < 0.05, comparing in both their littermates WT and male *Nf1*^+/−^ mice). **d** However, the analysis of ultrasonic vocalizations calls emitted during juvenile social play test indicated that mutant animals vocalize more in the non-social context, while WT mice prefer to communicate during social interactions (*p < 0.05, ***p < 0.001, ****p < 0.001, comparing percentage of calls emitted in non-social context). Data are expressed as mean ± SEM, *n* = 6–29. Statistical significance was found by two-way ANOVA followed by Tukey’s multiple comparisons test

Next, we investigated sex differences in social interaction. A juvenile social play test was performed and, overall, all experimental groups also showed a tendency to spend more time in the social context, but female *Nf1*^+/−^ spent relatively more time in a social context than in a non-social context (Additional file 1: Fig. S4). We found that mutant female spent more time socializing than mutant males (*Nf1*^+/−^ female: 1383.624 ± 92.865 s, male *Nf1*^+/−^ 1017.419 ± 79.143, *p* = 0.0295) and even than their littermates (WT female: 871.714 ± 101.267 s, *p* = 0.0018, Fig. 6a). Together, with socializing time, *Nf1*^+/−^ female also performed more prolonged social interaction and again compared with both mutant males (*Nf1*^+/−^ female: 19.183 ± 2.538 s, male *Nf1*^+/−^ 9.816 ± 1.091, *p* = 0.0016) and their littermates WT (WT female: 9.091 ± 0.759 s, *p* = 0.0051, Fig. 6b). In agreement, an augmentation of the social preference index in mutant females compared with WT females (WT: 7.571 ± 15.570, *Nf1*^+/−^: 53.497 ± 8.628, *p* = 0.0179), and more important, compared with mutant males (14.274 ± 3.973, *p* = 0.0412, Fig. 6c). In addition, a significant interaction sex × genotype was observed in all these social measures (social time: *F* (1, 24) = 7.791, *p* = 0.0101; social interaction time: *F* (1, 43) = 6.572, *p* = 0.0139; social preference index: *F* (1, 21) = 6.332, *p* = 0.0201).

**Fig. 7:**
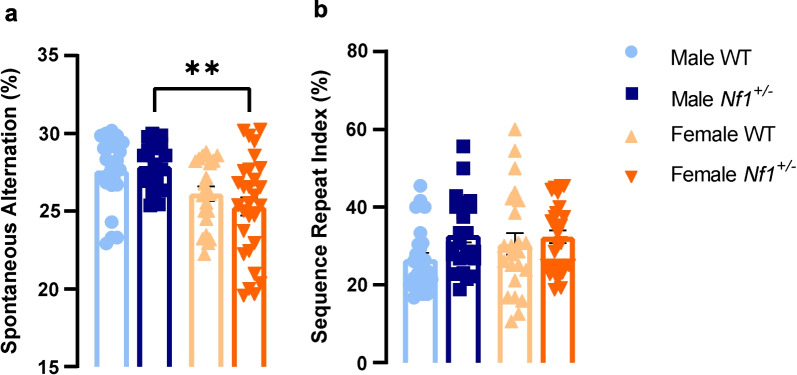
*Nf1*^+/−^ mutation alters repetitive behavior profile in juvenile male. **a** Spontaneous alternations measured during an object exploration test are less pronounced in mutant females, revealing than repetitive/restriction behavior is more present in mutant males (**p < 0.01, comparing with male *Nf1*^+/−^ mice). **b** No differences were observed between groups in sequence repeat index. Data are expressed as mean ± SEM, *n* = 3–29. Statistical significance was found by two-way ANOVA followed by Tukey’s multiple comparisons test

During social task, USVs emitted by animals were recorded. Our data demonstrated that there was a clear interaction between sex and genotype (*F* (3, 30) = 41.60, *p* < 0.0001, Fig. 6d). Indeed, both male and female WT mice vocalized more in a social than in a non-social context (male—social: 66.763 ± 6.133%, non-social: 33.238 ± 6.133%, *p* = 0.0270; female—social: 75.003 ± 8.333%, non-social: 24.998 ± 8.333%, *p* = 0.0006, Fig. 6d). Concerning mutant animals, despite of the fact that female *Nf1*^+/−^ spent more time socializing, they showed a higher percentage of calls in a non-social context (social: 14.136 ± 9.299%, non-social: 85.964 ± 9.259%, *p* < 0.0001, Fig. 6d), suggesting that a comprehensive view of social behavior requires different types of measures. Similar results were obtained in USV calls emitted by male *Nf1*^+/−^ mice (social: 9.090 ± 5.749%, non-social: 90.907 ± 5.751%, *p* < 0.0001, Fig. 6d). These data revealed that the *Nf1*^+/−^ mutation induces a reluctance for communication in social contexts and suggest that vocalization measures more directly reflect what is observed in humans. Furthermore, results from the novel object exploration test show that males display slightly more repetitive behaviors with a clear sex effect (*F* (1, 93) = 16.86, *p* < 0.0001). Indeed, our data revealed that *Nf1*^+/−^ females compared with *Nf1*^+/−^ males exhibit significantly less repetitive behavior, based on a spontaneous alternation measure (male: 27.88 ± 0.3155%, female: 25.30 ± 0.594%, *p* = 0.0015, Fig. 7a). However, the sequence repeat index showed no differences between groups (Fig. 7b).

## Discussion

The influence of biological sex on phenotypes of brain diseases is becoming increasingly recognized [23, 35]. In particular, several studies have shown the importance of biological sex in both normal brain maturation as well as in neurodevelopmental disorders. These disorders have a difference incidence in males and female, and the same holds true for the prevalence of symptoms and the response to therapeutic agents [36-38]. Concerning NF1, it has been further suggested that sex is determinant in both the early development and complications of the disease [17]. Diggs and collaborators described that males, both human and mice tend to develop cognitive impairments, whereas females tend to present a higher incidence of optic-gliomas [17]. Since majority of studies have been focused on males, more information is needed to understand the real impact of biological sex on NF1 symptoms, specifically in neurotransmission and cognition. Moreover, understanding sex differences will allow to better design diagnostic instruments and sex-tailored therapeutic approaches to NF1 and other ASDs.

For the first time, our study revealed that the neurobehavioral profile in *Nf1*^+/−^ is sex-dependent with surprising cross-species similarity with differential clinical phenotypes observed in male and female autistic patients [39]. We detected that mutant males tend to develop a more impaired cognitive profile, showing spatial memory deficits and more repetitive behavior. On the other hand, *Nf1*^+/−^ females developed internalizing symptoms, namely, anxiety-like behaviors. While both sexes presented difficulties in social communication, our data demonstrated that *Nf1*^+/−^ female show a preference for social contexts. Similar to our results, autistic girls have shown associated problems such as anxiety and tend to produce normative gaze patterns which can help them during social interaction contexts [40-42], while boys showed more externalizing disorders, such as hyperactivity, aggression and impulsivity [40, 42, 43]. This ability of females to mask their autistic traits through, for example, diminishing their social hurdles, has been called a “camouflaging”-type behavior, which adds to the difficulty of diagnosis in females [41, 44]. Here, we postulate that the *Nf1*^+/−^ mouse model can be an important resource for the investigation for new diagnostic tools and therapeutic outcome measures tailored to sex.

Previously, we reported that adult *Nf1*^+/−^ males display a region-specific E/I imbalance with both pre- and post-synaptic changes [15]. Our study suggested that each brain region may develop distinct compensatory mechanisms to bypass E/I imbalance [15]. Now, our data revealed the complexity of NF1 disorder by proving that E/I is not only region- but also sex-dependent. In fact, we found that at juvenile age, *Nf1*^+/−^ females display an increase in hippocampal GABA neurotransmitter and GABA(A) receptors levels. Surprisingly, mutant females exhibit a relative increase in spatial memory performance resembling the human phenotype, where ASD female patients demonstrate better executive functions and enhanced attention to faces [39]. On the contrary, mutant males showed an imbalance characterized by a decreased hippocampal levels of GABA(A) receptors together with an increase in brain weight and hippocampal volume and thickness. Interestingly, these characteristics were presented only in juvenile males. Accordingly, our group found that adult *Nf1*^+/−^ male have larger hippocampal volume than wild-type (WT) mice, a phenotype which is related with social cognition and memory [33]. These results are largely consistent with structural studies in adult NF1 patients, where increases in white matter and grey matter volumes were frequently observed [45, 46]. Larger volumes of thalami, right caudate and middle corpus callosum have also been reported in children with NF1 [47, 48]. Unfortunately, neither of these human studies discriminated between male and female. The same is true of the studies published so far, which aim to assess neurochemical differences in NF1 patients as well as in the animal model. Our group demonstrated that children and adolescents showed reduced GABA levels in the medial frontal cortex, occipital cortex and frontal eye fields [13, 14], although no sufficient statistical power was present to test for sex differences.

Previous works provided support that imbalance of the E/I ratio is associated with learning and cognitive deficits [49]. Many authors reported that NF1 individuals developed learning disabilities notably for reading, spelling and mathematics [50, 51]. However, a few studies demonstrated distinction between sexes. Accordingly, both human and mouse studies demonstrated that NF1 males showed more propensity to cognitive impairments [16, 17]. Furthermore, neurocognitive assessment of children and adolescents with NF1 have shown impairments in spatial working memory [52]. Ullrich and colleagues have also demonstrated deficits in visual–spatial learning in NF1 individuals using an arena maze, a virtual environment task that has been developed as a human paradigm to the Morris Water Maze, which is used to evaluate spatial learning in animal models [53]. In addition, it was corroborated that impairments in spatial memory was only a characteristic of male NF1 patients [17]. In agreement, we showed that male *Nf1*^+/−^ mice have spatial memory deficits. On the other hand, mutant female exhibited better spatial memory as compared with WT females. It is well-known the hypothesis of “peaks and valleys” of performance in individuals with ASD, both in terms of performance and brain activity. Indeed, we recently published that ASD adolescents during a social task reveals simultaneous hyperactivation across social, executive, and saliency circuits, and a reduction in activation in the parahippocampal gyrus [54].

A very recent report demonstrated sex differences in autistic social-communication behavior in children with NF1. The authors found that males exhibited greater social communication deficits relative to females [55]. Accordingly, in the juvenile NF1 mouse model we found that female spent more time socializing and in social interaction. This would apparently suggest a peak or at least preserved performance but the study of vocalizations provides a distinct insight. This technique suggested that both male and female have an impairment on social communication, with greater USV calls emitted in non-social context. The existence of contradictory studies may, therefore, depend on the interpretation of the behavioral measure used. Indeed, it was demonstrated that children with NF1 had significantly less ability in social domains, such as cooperativeness and assertiveness, than their unaffected siblings, but without sex differences [56]. These results were consistent across multiple sources obtained from parents, teachers and self-ratings made by children [48]. Again, one can postulate that in *Nf1*^+/−^ mouse a “camouflaging”-like behavior exists in females, since they were performing a “normalised” behavior under social context. In fact, autistic women exhibited higher adaptive mental processes to reach personal goals [57] and production of normative gaze patterns during social interaction contexts [58], rendering diagnosis of their condition difficult.

In agreement with clinical features of NF1 children [55], mutant female mice display a more anxious status than male. However, we observed that male *Nf1*^+/−^ mice tend to have a more repetitive behavioral pattern. Accordingly, repetitive/restrictive behavior in females are less frequent [59] and less based on objects [60]. Previous studies further stated that NF1 male patients tend to develop externalizing problems, such as hyperactivity and impulsivity [61].

## Perspectives and significance

Overall, our work emphasizes the influence of sex on the evolution of autistic traits/behaviors in NF1. For the first time, we showed that female *Nf1*^+/−^ mice display an “camouflaging”-type behavior mimicking women autistic traits. The mutant females presented better memory and social performance, in spite of exhibiting high levels of anxiety and a hippocampal E/I imbalance.

There is a growing interest in studying the importance of biological sex in the diagnosis, prevalence and severity of brain disorders. The present study validated the crucial importance of considering biological sex for improvement of diagnostic tools and, even, therapeutic approaches for NF1 and other ASD-related diseases. Moreover, one can postulate that *Nf1*^+/−^ mouse models represent a good approach to explore sex-directed phenotypic differences that may help inform diagnostic approaches.

## Conclusions

For the first time, our work validated a mouse model of ASD for the study of female “camouflaging”-type behavior. We clearly shown that female *Nf1*^+/−^ exhibited peaks and valley of performance, when compared with males, with better memory and social performance and worse internalizing symptoms. These findings will give a new perspective for the study of sex-tailored diagnosis tools.

## Supplementary Information

**Additional file 1: Figure S1.** Representative image of novel object explorationtest with the different objects used and the position number for sequenceanalysis. **Figure S2.** Representative image of mice hippocampus labelledwith toluidine blue taken from the morphometric microscope. Detail images indicated the measures used to analyze thickness ineach hippocampal sub-region—CA1, CA3and DG. **Figure S3.** Localizationof the magnetic resonance spectroscopyvoxelin thehippocampus, as well as a MRS averageplots of WT and Nf1+/− mice, highlighting the peaks of GABA and glutamate. **Figure S4.** Female Nf1+/− display an increase in social behavior. During a juvenile social playtest, it was found that female Nf1+/− mice spent more relativetime performing social interactions.

## Data Availability

The data sets used during the current study are available upon reasonable request.
