# Supplementary material for: Neurobehavioral sex-related differences in Nf1+/− mice: female show a “camouflaging”-type behavior
Source: Biol Sex Differ. 2023 Apr 26;14:24. doi: 10.1186/s13293-023-00509-8 (PMC10131355; doi:10.1186/s13293-023-00509-8)
Supplement: Supplementary file 1 — Additional file 1: Figure S1. Representative image of novel object explorationtest with the different objects used and the position number for sequenceanalysis. Figure S2. Representative image of mice hippocampus labelledwith toluidine blue taken from the morphometric microscope. Detail images indicated the measures used to analyze thickness ineach hippocampal sub-region—CA1, CA3and DG. Figure S3. Localizationof the magnetic resonance spectroscopyvoxelin thehippocampus, as well as a MRS averageplots of WT and Nf1+/− mice, highlighting the peaks of GABA and glutamate. Figure S4. Female Nf1+/− display an increase in social behavior. During a juvenile social playtest, it was found that female Nf1+/− mice spent more relativetime performing social interactions. [file 13293_2023_509_MOESM1_ESM.pdf]

Additional file 1

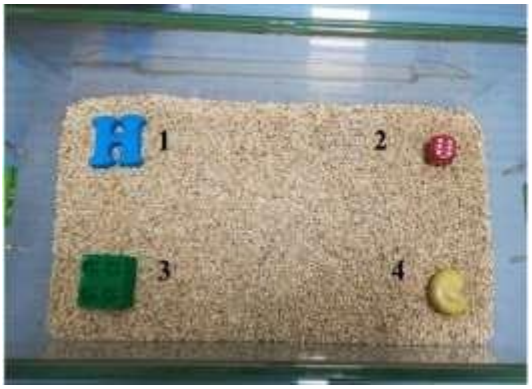

Figure S1. Representative image of novel object exploration test with the different objects used and the position number for sequence analysis.

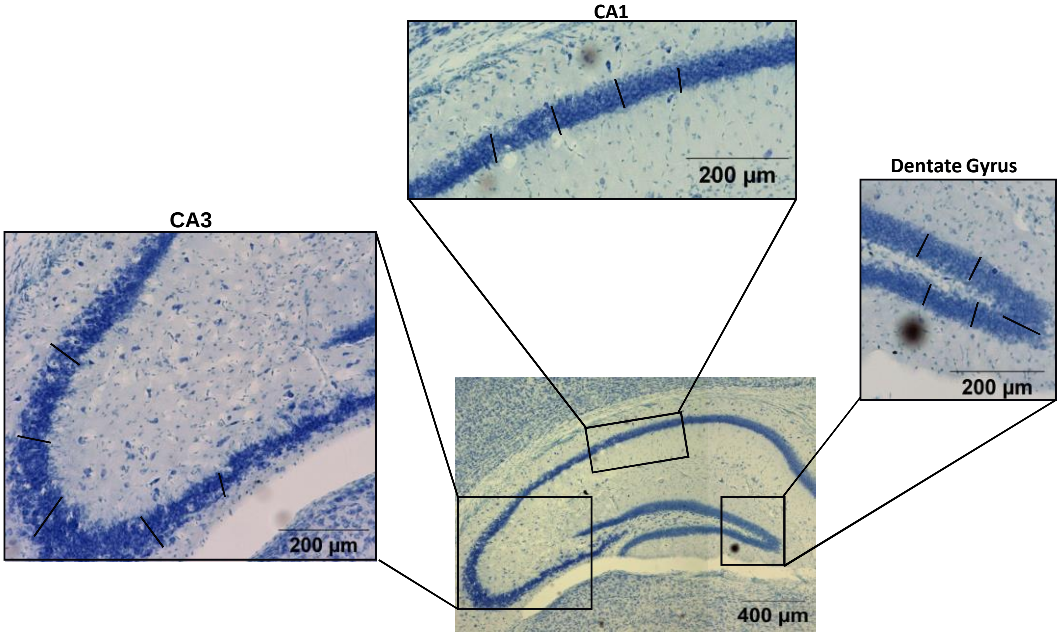

Figure S2. Representative image of mice hippocampus labelled with toluidine blue taken from the morphometric microscope (AX10, ZEISS, Germany). Detail images indicated the measures used to analyze thickness in each hippocampal sub-region - CA1, CA3 and DG.

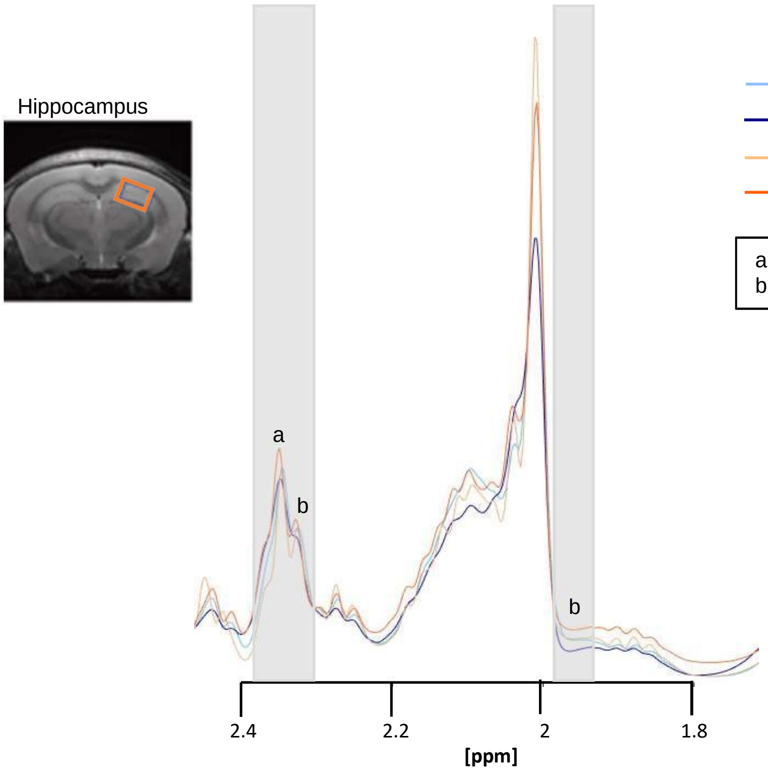

Figure S3. Localization of the magnetic resonance spectroscopy (MRS) voxel (orange square) in the hippocampus, as well as a MRS average plots of WT and Nf1+/- mice, highlighting the peaks of GABA and glutamate.

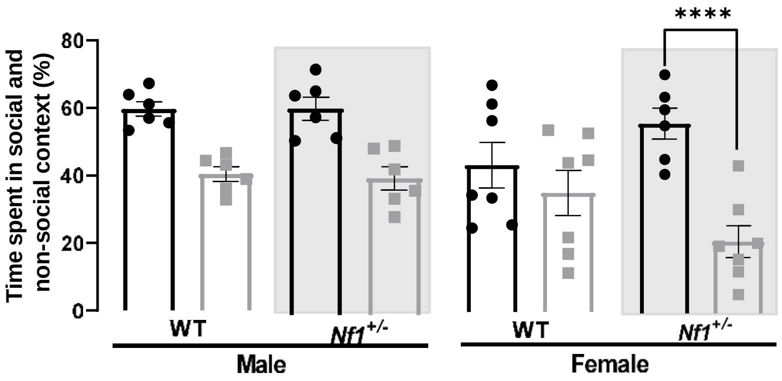

Figure S4. Female *Nf1*$^{+/-}$ display an increase in social behavior. During a juvenile social play test, it was found that female *Nf1*$^{+/-}$ mice spent more relative time performing social interactions. (****p < 0.001 by Mann-Whitney test, comparing with time spent in non-social context).
